# The Interactions of Insulin and Vitamin A Signaling Systems for the Regulation of Hepatic Glucose and Lipid Metabolism

**DOI:** 10.3390/cells10082160

**Published:** 2021-08-21

**Authors:** Guoxun Chen

**Affiliations:** Department of Nutrition, University of Tennessee at Knoxville, Knoxville, TN 37996, USA; gchen6@utk.edu; Tel.: +1-865-974-6257; Fax: +1-865-974-3491

**Keywords:** insulin, diabetes, vitamin A, retinoic acid receptor, retinoid X receptor, primary hepatocytes, glucose, lipogenesis

## Abstract

The pandemics of obesity and type 2 diabetes have become a concern of public health. Nutrition plays a key role in these concerns. Insulin as an anabolic hormonal was discovered exactly 100 years ago due to its activity in controlling blood glucose level. Vitamin A (VA), a lipophilic micronutrient, has been shown to regulate glucose and fat metabolism. VA’s physiological roles are mainly mediated by its metabolite, retinoic acid (RA), which activates retinoic acid receptors (RARs) and retinoid X receptors (RXRs), which are two transcription factors. The VA status and activations of RARs and RXRs by RA and synthetic agonists have shown to affect the glucose and lipid metabolism in animal models. Both insulin and RA signaling systems regulate the expression levels of genes involved in the regulation of hepatic glucose and lipid metabolism. Interactions of insulin and RA signaling systems have been observed. This review is aimed at summarizing the history of diabetes, insulin and VA signaling systems; the effects of VA status and activation of RARs and RXRs on metabolism and RAR and RXR phosphorylation; and possible interactions of insulin and RA in the regulation of hepatic genes for glucose and lipid metabolism. In addition, some future research perspectives for understanding of nutrient and hormone interactions are provided.

## 1. Introduction

In 2019, 463 million people had diabetes mellitus, which is one in eleven adults (20–70 years) worldwide [1]. The global burden of disease is concerning [2]. Type 2 diabetes mellitus (T2DM) accounts for about 90%, and this disease is due to the lack of insulin responses in the body, which is associated with profound changes of glucose and lipid metabolism [3]. Therefore, diet and lifestyle interventions play a key role in the prevention and management of T2DM.

Vitamin A (VA, retinol) was discovered more than a century ago as the first lipophilic factor from the diet that is essential for the gain of body weight in experimental animals [4]. VA’s role in vision, development and immunity, and its functional mechanisms have been revealed. The majority of these functions are mediated by the regulation of gene expressions [5,6,7,8]. More recently, it has been gradually realized that VA also plays a role in the regulation of glucose and lipid metabolism [9,10].

Insulin was discovered exactly 100 years ago due to its ability to lower blood glucose in patients with diabetes [11,12]. Since then, insulin has been found to regulate glucose and lipid metabolism in cells, and the alterations of these functions contribute to development of insulin resistance and T2DM [13]. These regulations of glucose and lipid metabolism can be partially attributed to insulin-regulated gene expression in the targeted cells [14,15,16].

As both insulin and VA regulate the expression of genes involved in glucose and lipid metabolism, it is reasonable to think that there is an interaction between insulin and VA signaling systems. The goal of this review is to discuss this interaction in the liver and hepatocytes. This may help the field to understand more about the role of VA in the regulation of glucose and lipid metabolism.

## 2. Insulin and VA

### 2.1. The History of Diabetes and Insulin

**Figure 1:**
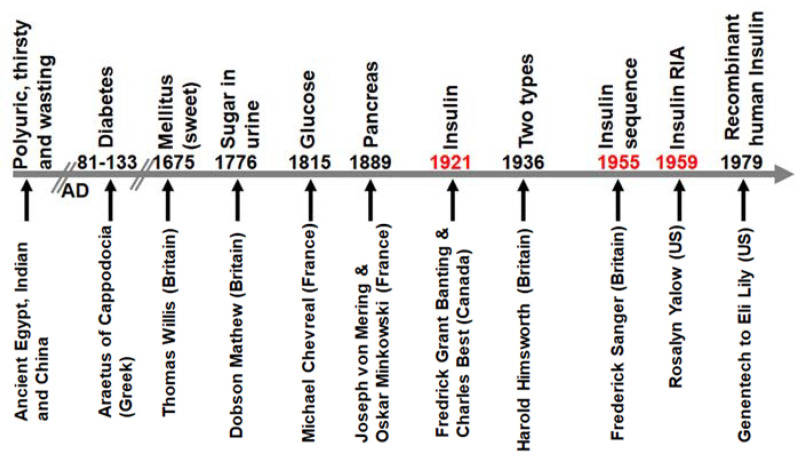
The pathway to understand and treat diabetes mellitus. The red font indicates the works that won the Nobel prize.

Diabetes has been known as a disease in human society for a long time, as shown in Figure 1. The polyuria symptom associated with diabetes was observed by people in ancient Egypt, Indian, and China [17,18]. Then, Aretaeus of Cappadocia described it as a polyuric wasting disease. Thomas Willis introduced the word “mellitus” to indicate the sweetness of the urine and blood of diabetic patients. Later on, Matthew Dobson observed sugar, and Michael Chevreul found glucose as the sugar molecule in the urine [17]. Joseph von Mering and Oskar Minkowski confirmed the link between the pancreas (an organ) to diabetes mellitus (a disease). Frederick Banting and Charles Best hypothesized that the pancreas has factors for regulating glucose and extracted a dog pancreas to test this activity, which resulted in the discovery of insulin in 1921 and the Nobel prize to Banting and McLeod in 1923 [11,12]. The use of insulin to treat patients with diabetes led Harold Himsworth to observe that there are two types of patients, the insulin-sensitive and insulin-resistant ones [19]. This is the beginning of the current type 1 diabetes and T2DM. Frederick Sanger, who had won Nobel prizes twice due to his contributions to protein and DNA sequencing, successfully sequenced the amino acid sequence of insulin in 1955 [20]. In 1959, Rosalyn Yalow, the second woman to win a Nobel Prize in Physiology or Medicine, developed the radioimmunoassay to accurately determine the blood insulin levels in humans, which confirms that patients with T2DM actually have high blood insulin levels, indicating insulin resistance [21]. With the development of recombinant DNA technology, human insulin was largely produced for the treatment of patients with diabetes [22].

The absorption of nutrients after feeding stimulates the secretion of insulin from the pancreatic β cells into blood. The rise of plasma insulin level signals the body to use and store nutrients in tissues and organs [3,23]. This is initiated after insulin binds to its receptor on the cell membrane, which contains receptor tyrosine kinase activity. The insulin receptor is a tetramer with two α subunits and two β subunits linked by disulfide bonds [24]. The binding of insulin to its receptor brings the two β subunits that are tyrosine kinases to proximity and allows them to phosphorylate with one another, a process that triggers signal transduction events and activates signal transduction cascades [25]. The signaling events include phosphorylation and dephosphorylation of proteins in cytosol and membranes and eventually change the activities of proteins and metabolic pathways in cells [13,25]. The activated insulin receptor β subunit tyrosine kinase phosphorylates insulin receptor substrates (IRSs) first [26]. The phosphorylated IRSs become a platform for the recruitment of other players, which transmits signals through protein–protein interactions and further modifications of downstream proteins [27], such as the regulatory subunits of phosphatidylinositol 3-kinase (PI3K), growth factor receptor-bound protein 2, and protein tyrosine phosphatase-2 [28]. After that, additional proteins in the signal network such as the PI3K/protein kinase B (PKB/Akt) pathway, GRB2/mitogen activated protein kinase (MAPK) pathway, and mammalian (mechanistic) target of rapamycin (mTOR) are activated [29,30,31,32]. The changes of these components in the signal transduction network eventually alter the metabolic homeostasis.

Insulin controls metabolic homeostasis at least in part by the regulation of gene expression [15]. Insulin treatment has been shown to regulate transcription through the insulin-responsive elements (IRE) on the promoter of the targeted genes involved in glucose and lipid metabolism [14,15]. For example, in the liver, insulin treatment affects the transcription of glycolytic, glycogenic, lipogenic, and gluconeogenic genes and, in turn, controls the energy metabolism [14]. For the hepatic glycolysis, insulin treatment induces the transcription of glucokinase gene (*Gck*), also known as hexokinase D [33,34]. However, the IRE of hepatic *Gck* has not been determined. On the other hand, proteins interacting with IRE at the promoters of other insulin-regulated genes have been reported. For example, members of the FoxO family of transcription factors have been shown to interact with IRE of some insulin-responsive genes involved in fuel metabolism and aging [35,36], etc. The mechanisms by which the PI3K/Akt and other signaling pathways regulate the activities of FoxO transcription factors have been conservative across species [35,36,37,38]. The current model of action is that insulin activates the PI3K/Akt signaling pathway and results in the phosphorylation of FoxO1, which leads to the attenuation of FoxO1-mediated transcription via nuclear exclusion as explained in [36].

For the hepatic lipogenesis, insulin specifically induces the transcription of sterol regulatory element-binding protein 1c gene (*Srebp-1c*) [39], which has been shown to be responsible for the upregulation of genes for lipogenesis [40]. The IRE of *Srebp-1c* is made of two liver X receptor element (LXRE) and one sterol regulatory element (SRE) in the promoter of *Srebp-1c* gene [41]. The development of the chronic metabolic diseases has been associated with profound changes of these genes’ expression, which have been attributed to metabolic changes [42].

### 2.2. VA and Its Signaling

VA (retinol) is a micronutrient needed to maintain the general health of a subject [5]. It must be obtained from dietary sources that contain molecules with VA activities in two forms, preformed VA (retinol and retinyl esters) and provitamin A carotenoids from animal and plant sources, respectively [43]. Carotenoids are converted into retinal, which is reduced into retinol in enterocytes and hepatocytes [44,45]. The body’s VA level is a dynamic balance of the dietary VA intake, body’s VA storage, and usage. This homeostasis is achieved through a network of enzymes and proteins responsible for VA digestion, absorption, transport, and catabolism [46]. VA’s physiological functions are mainly mediated by its metabolites, retinal that is involved in the vision cycle, and retinoic acid (RA) that regulates gene expressions [6].

Mammalian nuclear receptors are a super family of transcription factors with 48 members that regulate gene expression in response to the change of ligands [47]. The ligands, for their activations, cover a broad range of molecules such as thyroid and sex hormones, fatty acids and oxysterols, and bile acids and RA [47]. The general structure of a nuclear receptor includes a DNA binding domain, a ligand-binding domain (LBD), a N-terminal constitutive activation function 1 (AF-1), and a C-terminal ligand-regulated transcriptional activation function 2 (AF-2) [48]. The interactions of these domains among themselves and with other cofactors are critical for these transcription factors to modulate their target gene expressions [47,48,49]. RA is an agonist for retinoic acid receptors (RARs) and retinoid X receptors (RXRs) [7,50], which are two subfamilies of nuclear receptors [47]. The introduction of molecular cloning techniques allows the identification of isoforms of RARs (RARα, β, γ) and RXRs (RXRα, β, γ), which mediate RA’s ability to regulate gene expressions [49]. RXRs have been shown to act as a heterodimer partner for a variety of nuclear factors, and 9-*cis* RA has been thought of as the ligand for RXRs [47]. These transcription factors bind to the retinoic acid response elements (RARE) in the promoters of RA responsive genes. In addition to RARs and RXRs [51,52], hepatocyte nuclear factor 4α (HNF4α) [53], chicken ovalbumin upstream promoter-transcription factor II (COUP-TFII) [54], and peroxisome proliferator-activated receptor β/δ (PPAR β/δ) [55] have been reported to contribute to RA signaling [56].

### 2.3. The Impacts of VA Status on Body Weight and Metabolism

VA status has been shown to regulate body weight, glucose, and lipid metabolism in animal models [9,57]. An early study showed that the hepatic VA content in patients dead due to diabetes was elevated [58]. Depletion of the glycogen in the liver was observed in VA deficient (VAD) rats [59]. On the other hand, the hepatic glycogen levels were increased when rats were fed a diet with excess VA in the form of retinyl ester [60]. The reduction in body weight and the loss of total carcass fat in a rat fed a VAD diet have been reported as well [61]. We have fed Zucker lean (ZL) and Zucker fatty (ZF) a VA sufficient (VAS) or VAD diet for 8 weeks [62,63,64,65]. The 8-week protocol was the original protocol showing the need of VA to support rats to gain body weight [66]. We have observed the depletion of body fat in ZL rats and the attenuation of obesity development in ZF rats fed the VAD diet. As VA deficiency also results in a reduction in food intake, we have pair-fed the ZL and ZF rats with the same amount of calories [62]. The ZL and ZF rats fed the VAS diet with the same number of calories have higher body weight than those fed the VAD diet. The lowered body weight observed in VAD rats could not be explained solely by the reduction in energy intake [62]. We have shown that VA status also regulates the respiratory exchange ratio in ZL rats, indicating that VA plays a role in facilitating the anabolism in ZL rats [65]. Interestingly, VAD mice have triacylglycerol accumulation and low expression levels of genes for fatty FA oxidation in the liver, which is associated with downregulation of PPARα expression [67]. It appears that rats and mice respond differently to dietary VA deficiency. The underlying mechanism may be essential for us to understand the role of VA in the regulation of metabolism.

Retinaldehyde (retinal), the oxidized product of retinol, has been thought to act as an inhibitor for the PPARγ/RXR action in fat cells and improve insulin resistance in *ob/ob* mice [68].

In an attempt to understand the role of VA metabolism in the regulation of hepatic gene expression during the normal cycle of fasting and refeeding, the VAD and VAS ZL rats were fasted first and then refed a VAD or VAS diet for 6 h [65]. In this experimental setting, the only VA available to VAD rats is from the VAS diet during the refeeding process. We have shown that the VA from the diet for VAD rats and from the hepatic storage in VAS rats contribute to the induction of *Gck* mRNA and GK protein expression [65], which supports our original observation that retinoids synergize with insulin to induce *Gck* mRNA in primary hepatocytes [69]. In Ins2^Akita^ mice, a mode of type 1 diabetes due to the spontaneous mutation in the insulin 2 gene, the hepatic expression levels of *Cyp26a1*, *Rarb*, *Rdh10*, and *Rdh 13* are higher than that in the C57BL/6J control mice, which is associated with the increase in NAD+-dependent retinol dehydrogenase activities of microsome in the mutant mice, suggesting the interaction of VA metabolism and type 1 diabetes [70]. In C57BL/6J mice, a 16 h fasting but not a 24 h fasting reduces the hepatic levels of RA, but not retinyl esters and retinol [70]. On the other hand, the 6 h fasting is sufficient to reduce the hepatic *Gck* and *Cyp26a1* gene expression, suggesting the reduction in RA signaling [70]. We have reported that the hepatic expression levels of retinal dehydrogenase 1 mRNA and protein in ZF rats are higher than that in ZL [71]. All these results demonstrate that VA metabolism changes in normal and metabolic disease conditions, which may contribute to the regulation of hepatic gene expression.

### 2.4. The Effects of RA on Metabolism

Various isoforms of RA regulate activities of RARs and RXRs to control gene expressions [47]. As a VA metabolite, RA has been used as a drug and is a representative of VA functions. When patients with acne are treated with 13-*cis* RA (isotretinoin), some of them develop hypertriglyceridemia [72]. On the other hand, RA treatment has been shown to reduce body weight in diet-induced obese mice [73,74] and white adipose tissue weight in lean mice [75]. RA was thought to activate PPARβ/δ and RARs and to facilitate lipolysis and reduce fat accumulation in mature white adipocytes [73,74]. It is obvious that RA can regulate lipid metabolism in both human and animals.

The effects of dietary RA on insulin resistance in male normal C57BL/6J mice or genetically insulin-resistant KK-Ay mice and *ob/ob* mice has been investigated [76]. RA was blended into the diet at 50 mg/kg diet, which was used to feed KK-Ay mice and *ob/ob* mice for 4 weeks. The RA treatment reduces the fasting blood glucose in KK-Ay mice but increases it in *ob/ob* mice [76]. In this study, the effects of RA on the body weight and insulin resistance in C57BL/6J mice fed a higher-fat and high-fructose diet were investigated. C57BL/6J mice were first fed a higher-fat and high-fructose diet for 16 weeks, and then they were fed a diet without or with RA for an additional 4 weeks. C57BL/6J mice fed the normal diet for 20 weeks served as a control. Unfortunately, a control group with C57BL/6J mice fed the normal diet for 16 weeks and then fed the diet with RA was not included. Therefore, the impacts of dietary RA on the insulin levels and glucose metabolism in C57BL/6J mice fed a normal diet could not be determined in this experimental design [76].

RA (0.16 mg/day) treatment for 3 weeks has been shown to represses obesity and insulin resistance in C57BL/6Ntac mice fed a high-fat/high-sucrose diet for 16 weeks before treatment [73]. This is attributed to the activation of PPARβ/δ and RARs. On the other hand, overexpression of RARβ using recombinant adenovirus in 8-week-old C57BL/6J mice for 2 weeks results in the increase in fibroblast growth factor 21 production and hepatic fatty acid oxidation [77].

The human skeletal muscle expresses all three isoforms of RXR, but they are not related to insulin resistance, obesity, and type 2 diabetes [78]. RXRγ variations have been associated with elevated lipid levels in patients with T2D [79]. The 9-*cis* RA at 1µM was able to induce p85αPI-3K mRNA at 6 h and protein levels at 12 h in human myotubes [80]

The 13-*cis* RA is shown to reduce the expression level of angiotensin type 1 receptor (AT1) in rat liver epithelial cells and rat aortic smooth muscle cells through the activation of the Erk mitogen-activated protein kinase (MAPK) pathway [81]. This phenomenon is independent of glucose and insulin.

The plasma level of VA appears to be low in human patients with type 1 diabetes but elevated in those with T2DM [82,83]. VA is stored in pancreatic stellate cells [84]. RA treatment (0.5 mg/mouse intraperitoneally every other day) for 6 weeks is able to delay the onset of diabetes in NOD/Scid mice [85]. In alloxan-induced type 1 diabetic rats, RA treatment (0.20 mg/kg) for 17 days is able to reduce the blood glucose [86]. Both mouse and rat studies have attributed the benefits of RA treatment to the improvement of pancreatic β cell functions [85,86]. More studies are needed to understand the roles of VA in the control of pancreatic functions, as discussed in [84]. In addition, the RA effects may be attributed to the regulation of glucose metabolism [87].

Liquid chromatography linked to tandem mass spectrometry has been developed to compare the RA levels in patients with T2DM and aged matched controls [88]. The plasma levels of RA in T2DM patients are at about 1.38 ng/mL, which is significantly lower than 1.77 ng/mL in the control subjects [88]. Whether the difference reflects the differential RA production or degradation remains to the determined. Nevertheless, the activations of RA signaling pathways profoundly affects metabolism in humans and animals. The short-term and longer-term impacts on human health should be determined by well-designed clinical trials.

## 3. The Regulation and Activation of RAR and RXR on Metabolism

### 3.1. The Changes of RARs and RXRs in Metabolic Disease Models

In mice fasted for 48 h, *Rxrg* mRNA expression was rapidly induced after refeeding for 3 h and maintained for up to 12 h [89]. The hepatic *Rxrg* mRNA expression level in the fasted *db/db* mice is also higher than that in the control mice [89]. The refeeding induces the *Rxrg1* in the muscle and *Rxrg2* in the mouse liver [89].

On the other hand, reduction in RXR signaling appears to attenuate HFD-induce obesity and T2DM [90]. The intraperitoneal injection of a RXR antagonist HX531 at 10 μg/g body weight/day for 14 days shows anti-obesity and anti-diabetic effects in KKAy mice, which is associated with metabolic changes in adipose tissues, skeletal muscle, and the liver [90]. In addition, heterozygous PPARγ-deficient mice developed lipoatrophy, hyperglycemia, and insulin resistance when treated with HX531 in a HFD setting [90].

The transgenic mice with overexpression of RXRγ in the skeletal muscle have elevated glucose disposal due to the increase in the GLUT1 expression in the skeletal muscle [91].

Interestingly, the liver specific deletion of *Rxra* results in the reduction in food intake, elevations of body weight and fat, and improvement of glucose tolerance [92]. The plasma leptin and insulin-like growth factor 1 (IGF-1) levels are also increased in mice fed a regular diet or HFD [92].

The studies summarized in here show that the expression mRNA levels of *Rars* and *Rxrs* change in response to physiological changes such as fasting and refeeding in wild type and genetic mutant mice. In addition, alterations of *Rxrs* genes using transgenic overexpression and tissue specific deletion result in changes of lipid and glucose metabolism in mice. All these demonstrate the critical roles of *Rars* and *Rxrs* gene expression in the regulation of metabolism.

### 3.2. Targeting RARs and RXRs to Control Metabolism

Activations of RARs and RXRs have been involved in the regulation of various physiological functions. Since RXRs are heterodimer partners for multiple nuclear receptors such as RARs, PPARs, and liver X receptors (LXRs) [47], etc., the identification of specific ligands for the activation of nuclear receptors has been the focus in drug discovery [93,94]. For the treatment of metabolic diseases, the focus has been always to find specific agonists that help in improving certain physiological parameters. Specific agonists for the activation of RXRs have been developed.

UAB126, a novel molecule that has the activity of rexinoids, has been shown to ameliorate obesity, insulin resistance, hepatic steatosis, and hyperlipidemia in rodents [95]. This is achieved through the regulation of the hepatic genes that can be affected by the activations of PPARα and/or LXR in conjunction with RXR [95].

The activation of RARα via Am80 appears to attenuate the elevated β-oxidation and fat content in the heart of Zucker diabetic fatty rats, whereas activation of RXRs via LGD1069 resulted in body weight gain, hyperlipidemia, and fat accumulation in the heart [96]. In rat vascular smooth muscle cells, Am80 treatments increase the RARα expression levels and P-AKT-Ser473 via a PI3K-depedent manner [97].

Bexarotene (200 nM), an agonist of RXR, has been shown to induce the expression of Angiopoietin-like 4 in muscle cells, which leads to the reduction in lipoprotein lipase activity [98]. Another RXR agonist, LG100268 (20 mg/kg bw), treatment corrected hyperglycaemia and hypertriglyceridaemia after 15 days in *db/db* and corrected hyperinsulinaemia after 14 days in *ob/ob* mice, showing that the activation of RXR has potential for correcting metabolic disturbance in mice [99]. LG100268 treatment (10 mg/kg) for 2 weeks in *db/db* mice causes elevation of plasma alkaline phosphatase activity and hepatomegaly [100]. One study shows that LG100268 (30 mg/kg) treatment for 15 days reduces the blood glucose and triglyceride levels, and increases body weight in *db/db* mice but not in lean control mice [101]. Interestingly, another study shows that LG100268 treatment increases glucose uptake and insulin signaling in both lean and *db/db* mice [101].

The treatment of LG100268 or LG100324 at 20 mg/kg bw for 14 days caused ZF rats to eat less, reducing blood insulin level [102]. LG100268 (30 mg/kg) treatment for 6 weeks significantly reduced food intake and body weight gain in ZF rats [103]. The impact on food intake can be observed after LG100268 is administrated directly to the brain (30 µg/animal) via intracerebroventricular injection for 4 days [103].

LG100754, an RXR/RXR antagonist but not the RXR/PPARα agonist, treatment (100 mg/kg/d for 14 days) has shown to attenuate insulin resistance in *db/db* mice [104]. LG101506, an RXR heterodimer specific modulator, corrects hyperinsulinemia in ZF rats [105].

### 3.3. RAR Phosphorylation

Human RARα is phosphorylated at serine residues of the LBD and AF1 regions [106]. The ser 77 in the AF1 domain of human RARα is phosphorylated by cyclin-dependent kinase 7 (CDK7), which was thought to increase its activity [107]. The Ser 77 of RARα1 is phosphorylated by cyclin-dependent kinase 7 (CDK7) kinase, which is a subunit of transcription factor II H complex [108]. Ser 77 and Ser 79 of RARγ1 can be phosphorylated by P38 MAPK and CDK7, respectively [108]. CDK 7 also phosphorylates Ser 77 and Ser 79 of human RARγ1 [109]. The Ser 77 and Ser 79 of RARs are in the loop between helices 8 and 9 [108]. Phosphorylation of RARs by CDK7 appears to affect their interactions with cofactors, whereas their phosphorylation P38 MAPK, which can be activated by RA, facilitates their degradation. All these regulate the RAR mediated transcription [108].

In co-transfection experiments, RA treatments in COS-1 and F9 cells are shown to downregulate the PI3K/Akt pathway, which is associated with the increases in the phosphorylation of Ser 66 and Ser68 of mouse RARγ2 [110]. Co-transfection of dominant negative Akt construct results in the phosphorylation of mouse RARγ2 Ser 66, the degradation of its protein, and increase in its transcription activation [110]. In the neuronal differentiation of mouse ESCs, the p-Ser 68 of RARγ2 appears to rescue the differentiation process in cells without RARγ2 expression [111].

In non-small cell lung cancer (NSCLC) cells, Akt is constitutively active, which is shown to phosphorylate Ser 96 of human RARα, which is conservative in RARs and across species [112]. This serine residue is in the DNA-binding domain, and its phosphorylation does not affect the interaction of a RARE oligo with the RARα/RXRα heterodimer [112]. Interestingly, this phosphorylation appears to impair the RARα’s activity in a reporter gene assay [112]. Thr181, Ser445, and Ser461 residues of RARα are shown to be phosphorylated by c-Jun MAPK (JNK) in NSCLC cells [113]. The phosphorylation of these residues results in ubiquitin-mediated degradation of RARα, which is attributed to the low RARα expression in mouse lung cancer cells and insensitive to RA treatments [113].

Protein kinase A (PKA) phosphorylates RARα1 at Ser 369 and RARγ1 at Ser 371, which are in the loop between helices 9 and 10 [108]. The phosphorylation of Ser 369 of RARα does not initiate significant changes in the structure of the ligand binding domain [114]. The Ser 379 of mouse RARγ2 (corresponding to Ser 371 of human RARγ1) has been shown to be phosphorylated by Akt after the activation of Claudins-mediated activation of Src-family kinase/PI3K pathway in F9 cells, which are mouse testis epithelial cells [115]. This consensus motif of Akt (ammino acids 376 to 379 of mouse RARγ2) is conservative among three RARs of mouse and vertebrate, and this motif is also present in 14 of 48 members of human nuclear receptors [115]. The phosphorylation of this site is thought to increase RARγ’s activity [115].

As shown in Table 1, RXRs can be phosphorylated by several kinases and at AF1, DBD, and LBD. A variety of materials and methods have been used to confirm the site and domains of phosphorylation. These include tagged proteins, specific antibodies, immunoprecipitation and mutant constructs, and reporter gene assays. The impacts of phosphorylation can result in changes of RAR activity, protein stability, and interacting with other proteins. It is worth noting that RARs can be phosphorylated by Akt as summarized here in Table 1, and the PI3K/Akt pathway is also in the insulin signal cascade [29,30,31,32].

### 3.4. RXR Phosphorylation

IRS-1 and IRS-2 mediate insulin/IGF-1 signaling in brown fat [118]. The proteomic data of phosphoproteins obtained from genetic knock mice show that these two IRS proteins have complementary and distinct pathways that are associated with phosphorylation of mouse RXRα at Ser 22 [118]. This AF-1 serine is responsible for the ligand independent activation of transcription of RXRα [119]. The phosphorylation of Ser 22 of RXRα was shown to reduce the proliferation rate of mouse F9 testicular cells in the presence of RA [120]. In 9–11 week old male C57BL/6 J mice fasted overnight, the retro-orbital injection of 1 unit insulin for 5 min resulted in the elevation of phosphorylation of RXRα at serine 22 in brown adipose tissue [121]. However, the phosphorylation state was not changed after the mice were refed for 2 h or 6 h but was reduced in mice fed a high-fat diet (HFD) for 8 weeks [121]. Interesting, re-expressing wild type RXRα or a RXRαS22A mutant results in similar transcriptomic profiles, indicating that RXRα S22 phosphorylation is dispensable for adipogenesis in brown fat [121].

In triple negative breast cancer tissue, the expression of differentiation antagonizing non-protein coding RNA (DANCR), a long non-coding RNA, is upregulated [122]. This DANCR is shown to interact with RXRα and facilities the phosphorylation of RXRα at Ser 49 and Ser 78 via glycogen synthase kinase 3β (GSK3β) [122]. The knockdown of DANCR and overexpression of RARα resulted in the reduction in PI3K catalytic subunit alpha expression and phosphor-Akt level in DA-MB-231 cells, suggesting a role of DANCR in the regulation of Akt pathway and cell proliferation [122].

The overexpression of GSK-3β in hepatocellular carcinoma cells (HCC) reduces RARβ expression and induces the phosphorylation (Ser 78 and Thr 82 based on human sequence) level of RXRα (using anti-phospho-serine and threonine antibodies) via direct interaction, which is thought to disrupt the formation of dimer with RARα on RARβ promoter, reduce RXR/RAR-mediated transcription, and promote HCC cell clonal formation [123].

In COS-1 cells, RXRα is phosphorylated in multiple sites in the AF1 and LBD regions [124]. These phosphorylation changes do not appear to alter the transactivation activities of the RARα/RXRα heterodimers in reporter gene assays [124]

Mice with a phosphorylation deficient hRXRα at Thr167 (*Rxra*^T167A^) increased and demonstrated a reduction in the conversion of glucose to fatty acid lowered acetyl-CoA carboxylase expression in white adipose tissue and ATP citrate lyase in the muscle, but without any change in the liver [125]. This Thr 167 is phosphorylated by protein kinase C (PKC). Blood glucose is normal in the fed state and not reduced after fasting, which is not associated with any changes of hepatic genes in glucose and fatty acid metabolism [125]. Interestingly, significant changes in gene expression in the adipose tissue of the knock-in mice and the expression of Slc2a4 gene (GLUT4 gene) were elevated during fasting [125].

Human RXRα is also phosphorylated at Ser 260 by the RAS-RAF-MAPK pathway, which disrupts its interaction with the vitamin D receptor [126,127]. The serine 260 of human RXRα is phosphorylated by Ras-Raf-MAPK pathway [128]. The Ser 260 phosphorylation is also studied in the presence of phosphorylation of Thr 82 in 293 cells, which shows a reduced interaction with RARβ [129].

It appears that RXRα can be phosphorylated by multiple kinases and at multiple sites in different domains, as shown in Table 2. The phosphorylation of Ser 22 is linked to IRS-1 and IRS-2 [118], and its phosphorylation appears to mediate RA action [119,120,121]. GSK3β phosphorylates Ser 49, Ser 78, and Thr 82 of RARα in order to regulate it activity [122,123] In addition, MAPK seems to be responsible for the Ser 260 phosphorylation, which reduces the interaction of RARα and VDRβ or RARβ [126,127,128,129]. Insulin signal cascade has been shown to affect these signal pathways [118]. It indicates that there is a link between insulin and VA signaling pathways.

## 4. Insulin and RA Interactions for Regulating Gene Expression in Hepatocytes

The daily feeding cycle results in the rise and fall of nutrient fluxes and hormonal levels in the body, which results in the observation that components in the diets may modulate insulin action and can cause insulin resistance [130]. Data from multiple randomized clinical trials have shown that reducing the nutrient intakes of patients with T2DM through bariatric surgery efficiently lowers blood glucose with a reduction in body weight at the same time, demonstrating the impact of dietary nutrients in the T2DM development [131,132,133,134,135,136,137,138]. It is reasonable to hypothesize that a dietary nutrient probably modulates insulin actions in the liver.

The author’s lab started to investigate the interactions of insulin and VA signaling systems in order to understand the etiology of T2DM, which is summarized in this section. Hepatic lipid metabolism plays a role in the development of insulin resistance [139], which is in part attributed to the changes of insulin-regulated gene expression [15]. The study of insulin-induced *Srebp-1c* transcription suggests that endogenous metabolites generated upon insulin stimulation probably activated the dimer of LXRs/RXRs interacting with the IRE on its promoter [41]. To prove this hypothesis, the author’s lab began to investigate the effects of endogenous lipophilic molecules on the insulin-regulated gene expression in primary rat hepatocytes [69,140]. This is achieved by extracting lipophilic molecules from the rat liver and analyzing their impacts on the expression of *Pck1* [140] and *Gck* [69] in primary hepatocytes, which are the two insulin-regulated genes for the hepatic gluconeogenesis and glycolysis, respectively. First, we found that the lipophilic extract from the rat liver tissue induces the transcription of *Pck1* in primary hepatocytes [140].

Interestingly, this lipophilic extract also contains an activity that can synergize with insulin to induce the transcription of *Gck*, and the active molecules are identified as retinol and retinal [69]. Retinoids, retinol, retinal, and RA are able to synergize with insulin to induce *Gck* expression in primary hepatocytes. The activations of RARs and RXRs using their respective agonists mimic the RA responses and synergize with insulin, showing the interactions of insulin and RARs and RXRs signaling pathways [69]. Later, we identified the RARE on the promoter of hepatic *Gck* gene, which is also a binding site of HNF4α and COUP-TFII [56]. When RARα is overexpressed using recombinant adenovirus, the *Gck* expression is induced dramatically in the presence of RA, which is further induced by more than 2,000-fold in the presence of both RA and insulin, showing the interaction of RARα and insulin [56]. Overexpression of RXRα shows the same pattern of *Gck* regulation as the β-gal control group. HNF4α overexpression blocked both the RA and insulin effects. Interestingly, COUP-TFII overexpression only reduced the basal *Gck* expression and blocks the RA but not insulin effects, showing the dynamic interaction of insulin with transcription factors associated with the RARE in the hepatic *Gck* promoter [56].

We went back to study the effects of retinoids on *Pck1* expression in primary hepatocytes [141]. Retinoids induce the transcription of *Pck1* in primary hepatocytes by using one of the two previously identified RAREs in the *Pck1* promoter [141]. The presence of RA raises the insulin-suppressed *Pck1* gene expression to a level similar to the level of the control group without any treatment, suggesting the impairment of insulin action [141]. On the other hand, RARα overexpression suppresses *Pck1* gene expression in the absence of RA and promotes its expression in the presence of RA and RA + Insulin [56].

We also found that retinoids synergize with insulin to induce *Srebp-1c* gene expression in primary hepatocytes [142]. This is mediated by the activation of RXRs as LG100268, a RXR specific agonist, synergizes with insulin to induce *Srebp-1c* expression [142]. However, in the presence of insulin, activation of RARs through TTNPB, an RAR specific agonist, significantly reduces *Srebp-1c* expression in the absence or presence of LG100268 [142]. These results show the interactions among insulin, RXRs, and RARs in primary hepatocytes. The RAREs are the two liver X receptor elements that mediate insulin-induced *Srebp-1c* transcription [41]. It is worth noting that LXRs form heterodimers with RXRs and play important roles in the control of lipid metabolism [47].

We also show that RA synergizes with insulin to induce glucose usage in L6 muscle cells [143]. This is due to the elevation of glycogen synthesis in the presence of RA and insulin. Activation of RARs using TTNPB, but not RXRs using LG100268, results in a synergistic effect with insulin [143]. This result indicates that the interaction of RA and insulin signaling pathways also exists in the muscle cells.

## 5. Conclusions and Future Perspectives

The functional mechanisms and roles in the physiology of both insulin and VA have been studied extensively for the past century. These have allowed us to understand more about the nutritional and hormonal regulations of glucose and lipid metabolism. Now, VA’s role in the regulation of glucose and lipid metabolism has been gradually revealed. Both VA status and RA treatments have shown their effects on metabolism in animal models. As mediators of RA and VA signals, RXRs and RARs are responsible for a variety of physiological functions, including the regulation of metabolic homeostasis. They have been targets for pharmaceutical interventions for metabolic diseases, such as obesity and diabetes. Some of the target genes of RARs and RXRs are also those regulated by insulin signaling pathways. There are interactions of insulin and RA in the regulation of some of the gene expressions in the liver cells, as shown in this document. On the other hand, RARs and RXRs are phosphorylated by a variety of kinases, some of which are also players in insulin signaling cascade. Therefore, whether insulin modifies the activities of RARs and RXRs is something that is deserved to be revealed. Given the current pandemic of obesity and T2DM, it is imperative to understand the roles of RARs and RXRs in mediating insulin actions and their interactions.

The following areas deserve to be investigated in order to better understand the interaction of insulin and RA signaling systems: (1) The responsible nuclear receptors mediating the effects of VA status and RA signaling in physiological and pharmacological settings, respectively. RA has been used as the mediator of VA signaling. However, it may not be useful to study the dampening or disappearing of VA signals at the times or spaces where the VA signal is not needed or should be suppressed. (2) The RAREs are also convergent points of hormonal and nutritional signals. It may be helpful to identify those RAREs of the genes affected by both insulin and VA. These RAREs are places for the associations of transcription factors other than RARs and RXRs and cofactors. Whether the insulin signaling system regulates the dynamic interactions of these RAREs with transcription factors and cofactors remains to be determined. (3) The open questions of whether insulin mediates phosphorylation of any of the sites in RARs and RXRs and what are the underlying mechanisms and physiological functions remain to be addressed. Certainly, the grand question is always how hormonal and nutritional signals converge at a gene and a protein and, in turn, influence the short-term and long-term health outcome.

Evidence has been gradually accumulated to show that VA status and metabolism contribute to the regulation of glucose and fat metabolism [9,10]. Whether the onset of T2DM or development of insulin resistance affects VA metabolism and its signaling pathways and whether alterations in VA metabolism facilitate or prevent the T2DM are future directions to be explored for revealing the interactions of VA and insulin actions. In addition, the use of VA supplementations in patients with T2DM should probably be monitored closely and frequently in order to evaluate the outcomes, which may benefit their quality of life. For drug development, pathways responsible for VA metabolism and dynamical activation or suppression of RA signaling at the promoters of genes involved in the control of glucose and lipid metabolism in different tissues and cells may be potential targets. For example, the use of RA as a drug in humans results in the development of hyperlipidemia [144]. Clinical trials are probably needed to determine whether suppression of endogenous RA production will be helpful for the control of hyperlipidemia, which may curtail insulin resistance. In addition, the development of VA deficiency is associated with the reduction in food intake in both wild type and ZF rats [62,64]. The question of whether there are any interactions of VA and insulin in the brain is still open. It appears that targeting the brain VA metabolism may be an option for controlling T2DM development. Nevertheless, more clinical studies are needed to evaluate the interactions of VA and insulin signaling systems, which may eventually help the patients with T2DM.

## Figures and Tables

**Table 1:** Sites, materials or methods, domain, and responsible kinases for RAR phosphorylation.

Proteins	Site	Materials	Methods	Doman	Kinase	Citations
Human RARα	Ser 77	In vitro labeling	Activity assay	AF1	Cyclin-dependent kinase 7	[107]
Human RARα	Ser 96	FLAG-tagger RARα, and GST-RARα	Immunoprecipitation and in vitro kinase assay	DBD reduced activity	Akt	[112]
Human RARα	Ser 369	Synthetic phosphopeptides (not specified sequence)	Mouse monoclonal	LBD	PKA, and MSK1	[116,117]
Human RARα	Thr181, Ser445, and Ser461	Recombinant GST-RARα	In vitro kinase assay	Multiple increases in degradation	JNK	[113]
Human RARγ1	Ser 77 and Ser 79	Whole cell labeling	Immunoprecipitated	AF1 modulates AF1 activity depending on constructs	CDK7	[109]
Zebrafish RARα	Ser 72 (Human Ser 77)	EEMVPSSPS(p) PPPPPRVYKPC	Mouse monoclonal	N-terminal proline-rich domain	CDK 7	[106]
Mouse RARγ	Ser 66 and Ser 68	Synthetic phosphopeptides	Rabbit polyclonal	AF-1 Caused degradation	P38 MAPK	[109,110,111]
Mouse RARγ2	Ser 379 (human 371)	Mutant constructs	Immunoprecipitation	LBD Stimulates activity	Akt	[115]

Note: AF1, activation function 1; CDK7, cyclin-dependent kinase 7; DBD, DNA binding domain; JNK, c-Jun N-terminal Kinase; LBD, ligand-binding domain; MAPK, mitogen-activated protein kinase; MSK1, Mitogen-activated and stress-activated protein kinase-1; PKA, protein kinase A; RAR, retinoic acid receptor.

**Table 2:** Sites, materials or methods, domain, and responsible kinases for RXR phosphorylation.

Proteins	Site	Materials	Methods	Domain	Upstream Kinase	References
Mouse RXRα	Ser 22	Mutant constructs and cell lines; SSLNS(p) PTGRGS phosphopeptide	Western blots; anti-phosphor antibody	AF1 required for RA-induced activity	Proline-dependent kinase	[119,120,121]
Human RXRα	Ser 49 and Ser 78	synthesized peptide	Phospho-RXRA (Ser260) ( AB_2663160)	AF1 decreases its inhibition to PI3KC promoter	GSK3β	[122]
Human RXRα	Ser 78 and Thr 82	Anti-phosphor antibodies, GST-RXRα	Immunoprecipitated	AF1 decrease interaction with RARα	GSK3β	[123]
Mouse RXRα	Ser 61, Ser 75, Thr 87 in AF1, and Ser 265	Mutant constructs	Immunoprecipitation and in vitro kinase assay	AF1 and LBD	JNKs	[124]
Human RXRα	Thr167	CKGFFKR-pT- VRKDLTY, human RXRα knock-in mice (RxrαT167A)	Phosphor specific antibody	DBD effects depending on the promoter contexts	PKC	[125]
Human RXRα	Ser 260	Anti-phosphor antibodies Mutant constructs	Immunoprecipitation Immunofluorescent	LBD decreases interaction with VDR or RARβ	Ras-Raf-MAP kinase	[126,127,128,129]
Human RXRα	Thr 82 and Ser 260	Mutant constructs	Immunoprecipitation, Western blot	AF1 and LBD decreases interaction with RARβ	MAPK	[129]

Note: AF1, activation function 1; DBD, DNA-binding domain; GSK3β, glycogen synthase kinase 3β; JNK, c-Jun N-terminal Kinase; LBD, ligand-binding domain; MAPK, mitogen-activated protein kinase; PKC, protein kinase C; RXR, retinoid X receptor.

## Data Availability

Not applicable.
